# Symptom improvement is associated with serum cytokine level change during RTMS tretament in patients with treatment resistant depression

**DOI:** 10.1192/j.eurpsy.2021.1315

**Published:** 2021-08-13

**Authors:** J. Lazary, M. Elemery, S. Kiss, L. Pogany, G. Faludi

**Affiliations:** 1 Department Of General Psychitary B, Nyírő Gyula National Institute of Psychiatry and Addictions, Budapest, Hungary; 2 Janos Szentagothai Neuroscience Doctoral School, Semmelweis University, Budapest, Hungary

**Keywords:** rTMS, TRD, Depression, noninvasive brain stimulation

## Abstract

**Introduction:**

Introduciton: Repetetive transcranial magnetic stimulation (rTMS) is an effective and safety noninvasive technique for treatment of major depression disorder (MDD). There is a body of increasing evidences on the potential molecular mechanisms underlying its effectivity even in case of treatment resistant depression (TRD), however, the exact mechanism is still not clarified. Among multiple biological systems, inflammation can be a target of rTMS in MDD (Tian et al. 2020; Tateishi et al. 2020).

**Objectives:**

Here we analysed serum cytokine levels in TRD before and after rTMS interventions.

**Methods:**

We used bilateral stimulation (15Hz for left DLPC and 1Hz on the right side) in 18 patients with TRD (5 men and 13 women; mean age=47.7±12.1 year) for 2x5 days. Blood samples were collected before the first (V1) and after the last inetrevntion (V2). Phenotypic changes were measured by Beck Depression Inventory (BDI), Beck Anxiety Inventory (BAI), Snaith–Hamilton Pleasure Scale (SHAPS), Insomnia Severity Index (ISI) and Stroop Color-Word Test (SCWT) modified by Golden. Inflammatory cytokines were assessed by ELISA assays.

**Results:**

Change of BDI and BAI scores between V1 and V2 is associated with difference of TNFalpha levels (p=0.043; adj.R2=0.42 p=0.011; adj R2=0.43). Decrease on SHAPS score has been depended on IL-6 level (p=0.027) and the interaction of TNFalpha and IL-10 (p=0.005; adj R2=0.63). Sleep disturbance and neurocognitive function was not associated with cytokine levels.

**Conclusions:**

Our results confirmed the association between depressive, anxious and anhedonia symptom improvement and inflammatory mechansims during rTMS treatment. The study was supported by the OTKA 151513 grant.

